# Coupled Multi-Parameter Optimization of a Differential Resonant Photoacoustic Cell with Impedance-Matched Pickup for Trace Acetylene Detection

**DOI:** 10.3390/s26216793

**Published:** 2026-10-27

**Authors:** Chao Gao, Jinxuan Song, Qiaogen Zhang, Zhicheng Wu

**Affiliations:** 1 State Key Laboratory of Electrical Insulation and Power Equipment, School of Electrical Engineering, Xi’an Jiaotong University, Xi’an 710049, China; gaochao@csg.cn (C.G.); xajdsjx@stu.xjtu.edu.cn (J.S.); hvzhang@xjtu.edu.cn (Q.Z.); 2 Electric Power Research Institute, China Southern Power Grid, Guangzhou 510663, China

**Keywords:** photoacoustic spectroscopy, resonant photoacoustic cell, coupled multi-parameter optimization, differential acoustic suppression, impedance-matched pickup, acetylene detection

## Abstract

The performance of a resonant photoacoustic (PA) cell depends on coupled geometric and acoustic parameters acting through the acoustic field, thermoviscous boundary losses, and the microphone load. Building on established analytical, numerical, and equivalent-circuit rules for PA-cell design, this work develops a system-specific co-optimization of a dual-tube longitudinal resonator for trace acetylene (C_2_H_2_) detection. Three aspects are integrated. First, beam–tube coupling and the buffer–tube length ratio are considered together; balancing antinode pressure, resonance frequency, and quality factor *Q* gives a tube radius of 3.25 mm and a buffer length equal to half the tube length, with a simulated three-dimensional photoacoustic conversion amplification of 1.6. Second, coupling through the shared buffers splits the first longitudinal mode into a 1597 Hz anti-phase mode and a 1621 Hz in-phase mode. The sensor is operated at the anti-phase mode, where differential extraction gives approximately twice the single-tube simulated amplitude while rejecting spatially coherent common-mode noise. Third, a transmission-line analysis identifies the low shunt impedance introduced by a conventional ducted microphone cavity and motivates a direct-insertion pickup. The fabricated cell has a C_2_H_2_ sensitivity of 24.6 mV/ppm ($R^{2}=0.9984$). At 300 s, the minimum Allan deviation is 9.4 ppb and the corresponding 3σ limit of detection is 28.2 ppb. A mixed-gas interference test performed with the same cell gives a C_2_H_2_ relative error of 4.09%.

## 1. Introduction

Dissolved gas analysis (DGA) in oil-immersed power equipment relies on the trace detection of characteristic gases, among which acetylene (C_2_H_2_) is the decisive indicator of high-energy discharge faults [1,2]. Online operation requires a detection limit at the parts-per-billion level over months of unattended running, a regime in which laser photoacoustic spectroscopy (PAS) is well matched because the signal scales with the absorbed optical power rather than with the dark floor of a photodetector [3,4]. A resonant cell concentrates the acoustic energy at a mechanical resonance, and most of the practical gain of a PAS instrument is set by the cell rather than by the source or the electronics [3,4]; the cell structure is therefore the key determinant of the achievable sensitivity.

PA-cell design has been studied extensively. Analytical design rules, numerical models, and experiments have quantified the effects of resonator length and radius, buffer dimensions, thermoviscous losses, optical-beam confinement, and gas composition in longitudinal and H-type cells [4,5,6,7,8,9]. Differential Helmholtz resonators provide common-mode acoustic rejection and have demonstrated sub-ppm gas detection [10]; equivalent-circuit and transmission-line approaches provide compact descriptions of Helmholtz, H-type, and differential cells [11]. Recent studies have also investigated T-type, trapezoidal, ellipsoidal, Y-sphere, hyperbolic, and cone–sphere resonators, as well as alternative transducers and multipass excitation [12,13,14,15,16,17,18,19]. Thus, the effects of multiple design parameters are well established in the literature; the remaining task for a particular architecture is to identify how those established effects interact under its specific optical, acoustic, and manufacturing constraints.

Non-conventional ring-shaped and toroidal resonators provide a complementary route. Fort et al. developed an optimized ring-shaped resonant cell with a folded acoustic path and compact footprint [20], while Panzardi et al. linked toroidal geometry to resonance frequency and *Q* using analytical relations, finite-element simulations, and experiments [21]. These closed-loop resonators possess continuous rotational symmetry and support azimuthal propagation without the terminated axial path of a cylindrical cell. In contrast, the present H-cell has discrete mirror and tube-exchange symmetries: two straight longitudinal tubes are coupled through shared end buffers. It retains a straight optical path and deliberately forms an anti-phase/in-phase longitudinal eigenmode pair for differential readout rather than a closed-loop azimuthal mode.

The contribution claimed here is therefore narrower than the introduction of multi-parameter PA-cell modeling. We apply established design principles within one system-specific sequence for a dual-tube longitudinal architecture, including beam–tube coupling, buffer dimensions, tube-to-tube modal coupling, and the local acoustic load of the microphone mount. The dependence of the cell constant on beam waist and resonator radius has been derived previously [8], and microphone–resonator impedance matching has been recognized in the lumped Helmholtz framework [22]. The modal behavior of dual-tube differential cells, including coupled in-phase and anti-phase eigenmodes, has also been reported experimentally [23,24]. The present work focuses on combining these considerations for the proposed geometry, quantitatively mapping the modal split and the two *Q* values against tube spacing, and integrating a direct-insertion pickup with the coupled cell.

This work applies that system-specific coupled design in three steps. First, the closed-form beam–tube coupling law $\lambda \left(R_{\mathrm{res}};\sigma \right)\cdot P_{\max}\left(R_{\mathrm{res}}\right)$ and the quarter-wavelength buffer condition jointly set the radial and longitudinal geometry under the constraints $f_{0}>1600$ Hz and Q>40. Second, the dependence of the dual-tube eigenfrequencies, the two *Q* values, and the differential and common-mode amplitudes on tube-center spacing is mapped quantitatively; the anti-phase mode is selected as the operating mode. Third, a transmission-line model of the resonance tube loaded by a microphone cavity motivates a direct-insertion microphone mount. The fabricated cell reaches a C_2_H_2_ sensitivity of 24.6 mV/ppm, a minimum Allan deviation of 9.4 ppb at 300 s, and a corresponding 3σ limit of detection of 28.2 ppb. Selectivity is evaluated with a prepared mixed-gas sample using the same cell.

## 2. Materials and Methods

**Figure 1:**
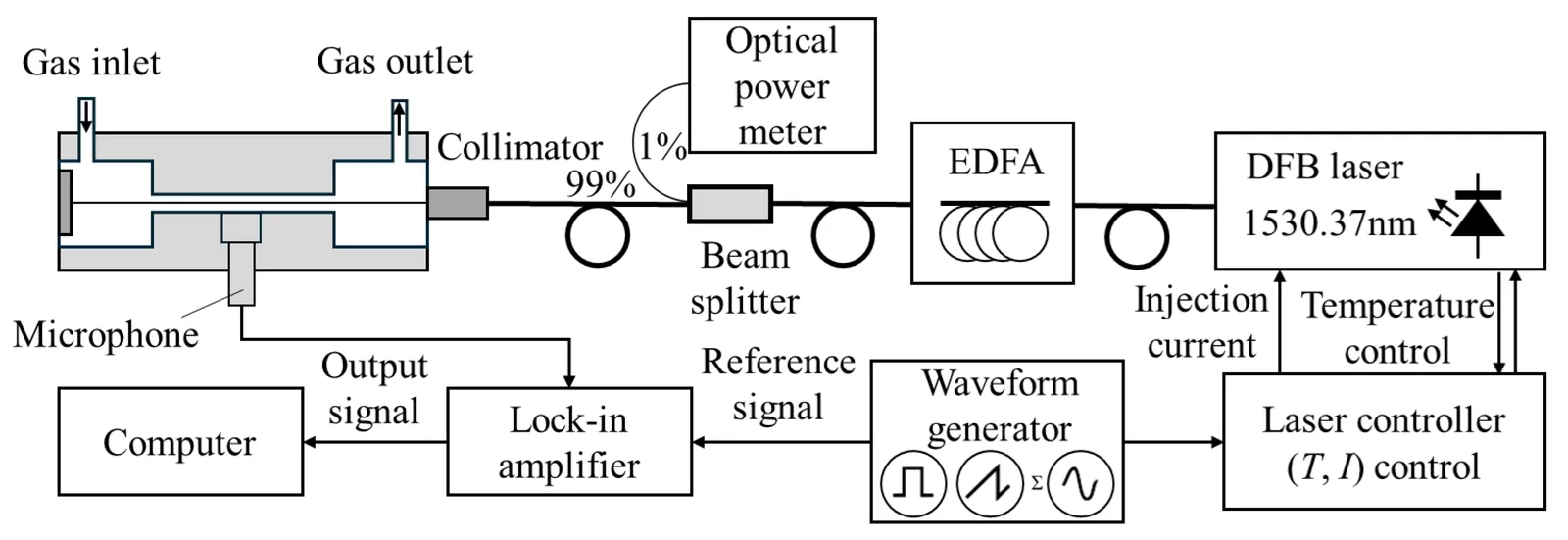
Schematic of the photoacoustic spectroscopy gas detection platform.

The coupled-parameter optimization developed in this work was evaluated on a resonant PAS platform built for trace C_2_H_2_ detection. The signal chain is shown schematically in Figure 1: a wavelength-modulated DFB laser (1530.37 nm) is amplified by an erbium-doped fiber amplifier (EDFA), split by a 99:1 fiber coupler, with 1% sent to an optical power meter (Thorlabs, Newton, NJ, USA; PM20CH) and 99% delivered through a fiber collimator into the resonant cell; the acoustic pressure at the antinode is converted by a microphone to an electrical signal, and a lock-in amplifier demodulates the harmonic of interest for concentration retrieval, using the modulation waveform generated by a waveform generator (Tektronix, Beaverton, OR, USA; AFG31000 series) as the reference. The platform comprises the optical source, the photoacoustic cell, and the acoustic detection electronics, together with a gas-handling module.

Optical source. The excitation source is a single-frequency distributed-feedback (DFB) semiconductor laser (Thorlabs, Newton, NJ, USA; model DFB1531, emission near 1531 nm). The acetylene line at 1530.371 nm in the near-infrared $\nu_{1}+\nu_{3}$ combination band (around 6500 cm^−1^) was selected over the stronger mid-infrared fundamental, because the combination-band region is comparatively free from the broadband absorption of oil vapors and environmental water vapor and is accessible to narrow-linewidth, low-drift DFB sources. The line was chosen because its absorption coefficient is the largest among the candidates and its neighbors are sufficiently far from the interfering lines of H_2_O, CH_4_, and CO_2_. The laser was driven by a low-noise current controller (Thorlabs, Newton, NJ, USA; model LDC200C) and temperature-stabilized by a TEC controller (Thorlabs, Newton, NJ, USA; model TED200C) in a closed PID loop with a fluctuation below 0.002 °C. The nominal 20 mW output was amplified by a 1531 nm single-mode erbium-doped fiber amplifier (Box Optronics, Shenzhen, China; model EYDFA-HP-BA-5330-SM-M31) and collimated by a fiber collimator (Thorlabs, Newton, NJ, USA; model CFS11-1550-APC). The measured $1/e^{2}$ waist diameter was approximately 2.15 mm. This measured optical diameter is distinct from the 1.6 mm effective Gaussian volumetric heat-source diameter used in the acoustic simulation below. The laser temperature and the sinusoidal modulation current were set to align the emission wavelength with the absorption-line center and to maximize the second-harmonic response, respectively, so that the platform operated in a regime balancing the signal-to-noise ratio and the resistance to temperature drift.

Photoacoustic cell. The cell was an H-type first-order longitudinal resonator terminated at both ends by buffer cavities of a larger cross-section, which confine the longitudinal standing wave to the resonator and suppress the stray background noise generated at the optical windows. The physical length of 80 mm and the diameter of 7.5 mm correspond to an effective length of approximately 86 mm and a target resonance near 2000 Hz; the buffer length equalled the resonator length and the buffer radius exceeded three times the resonator radius. A swept-frequency measurement of the fabricated cell yielded an actual resonance frequency of 1722.5 Hz and a quality factor of 18.72, in agreement with the first longitudinal mode predicted by a thermoviscous acoustic model; the residual discrepancy in *Q* was attributed to dead volumes and surface roughness omitted from the model.

Acoustic detection and demodulation. The acoustic pressure at the antinode was converted to an electrical signal by a miniature electret condenser microphone (Knowles, Itasca, IL, USA; model FG-23629-D65) of 2.57 mm outer diameter, small enough to be embedded in the cell wall without perturbing the resonant field. The microphone output, of the order of μV, was demodulated by a digital lock-in amplifier (Zurich Instruments, Zurich, Switzerland; model MFLI-500) with a 500 kHz bandwidth and a 120 dB dynamic range. Wavelength-modulation spectroscopy (WMS) was used, in which a low-frequency sawtooth current scans the laser across the absorption line while a high-frequency sinusoidal current modulates the wavelength; the second harmonic (2*f*) was extracted as the concentration-related quantity because it peaks at the line center and suppresses the background-absorption interference. The MFLI-500 supports simultaneous multi-demodulator operation, so the first and second harmonics were recovered synchronously from the same input channel, with the fundamental used for power normalization and the second harmonic for concentration retrieval.

Gas-handling module. The gas-handling module consisted of standard gases, four mass-flow controllers (Brooks Instrument, Hatfield, PA, USA; model 5850E R, 0–300 sccm, ±0.3% accuracy), a water-vapor saturator, and a gas manifold. High-purity nitrogen and oxygen (99.999%) and calibrated C_2_H_2_ standard gases in N_2_ (Shaanxi Longqing Gas Co., Ltd., Yulin, China) were mixed in the manifold according to the flow ratios. The saturator, formed by a constant-temperature water bath holding a saturated sodium chloride solution, supplied a fixed absolute humidity so that the effect of water vapor on the photoacoustic signal could be investigated under controlled conditions; with the bath at 35 °C, the equilibrium relative humidity above the solution was 74.87%, giving an absolute humidity of 4.27%.

## 3. Coupled-Parameter Design

**Figure 2:**
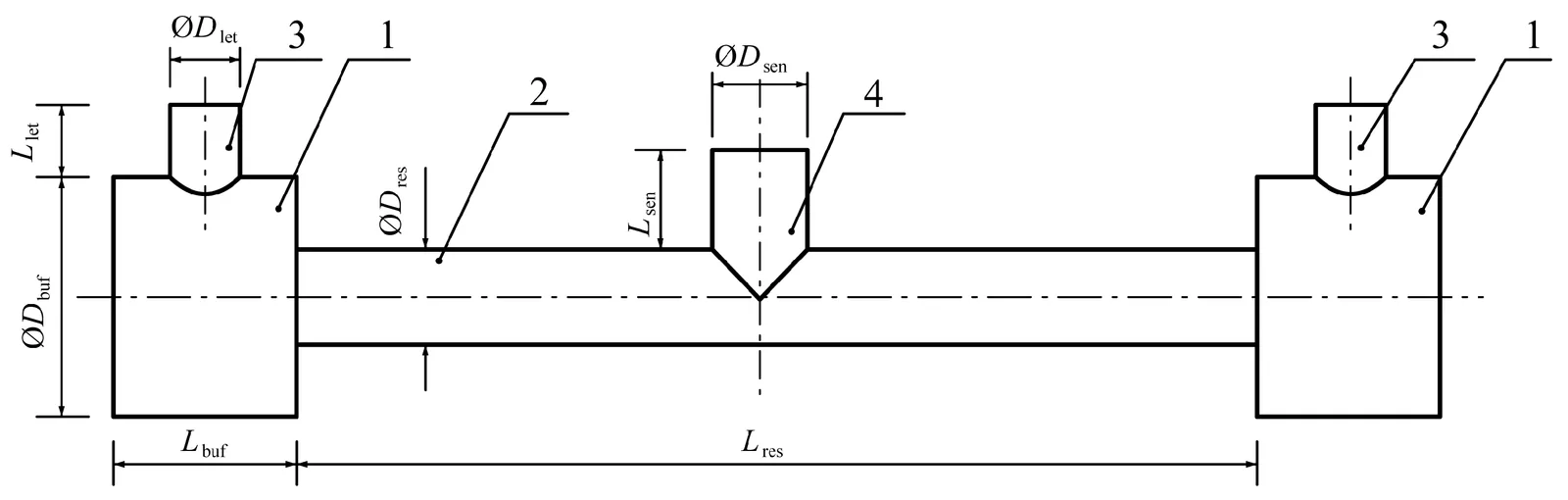
Schematic of the longitudinal resonant photoacoustic cell. Labels 1–4 identify the buffer cavity, resonance tube, gas port, and microphone mounting position, respectively. The dashed line marks the cell axis. In the dimension labels, L and D denote length and diameter, while the subscripts let, buf, res, and sen refer to the gas port, buffer cavity, resonance tube, and microphone mounting position, respectively.

Before entering the detailed design, the core question of this section should be made explicit: the design of a photoacoustic cell cannot be reduced to the independent selection of structural parameters. The performance of a first-order longitudinal resonator is characterized by three quantities—the antinode pressure (which sets the signal), the quality factor *Q* (which sets the resonance gain and the frequency selectivity), and the resonance frequency $f_{0}$ (which sets the operating point above the low-frequency noise floor). These three quantities depend jointly on the structural parameters (the tube radius, the beam waist, the buffer dimensions), and each structural parameter acts on more than one performance quantity. The tube radius constrains the energy deposition of the beam (smaller radii confine the beam more tightly, but a narrower tube raises the volume fraction of the viscous boundary layer and lowers *Q*) and, through the acoustic mass loading, sets $f_{0}$. The buffer defines the longitudinal acoustic boundary (and therefore $f_{0}$ and *Q*) and determines the transmission of external noise. The parameters must be solved as a coupled set, and this section proceeds from the radial coupling to the longitudinal coupling to the joint convergence. Figure 2 shows the optimized single-tube cell that the analysis in this section delivers.

### 3.1. Beam–Tube Diameter Coupling

**Figure 3:**
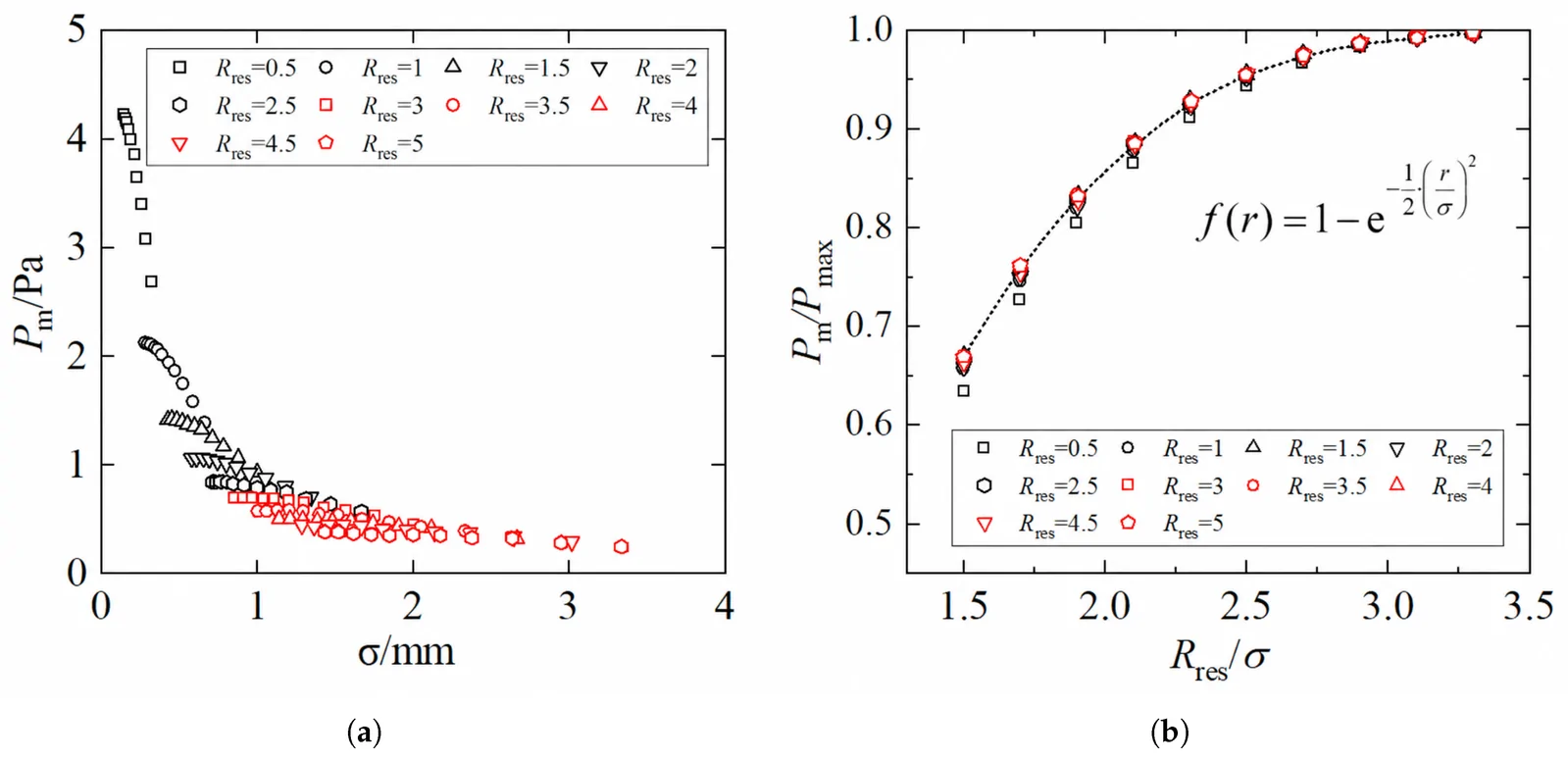
Beam–tube diameter coupling: (**a**) antinode pressure versus Gaussian heat-source width σ for different resonance-tube radii $R_{\mathrm{res}}$; (**b**) normalized antinode pressure versus $R_{\mathrm{res}}/\sigma$ for the same radius series.

A Gaussian beam of width parameter σ propagating through a tube of radius $R_{\mathrm{res}}$ deposits the fraction $\lambda \left(R_{\mathrm{res}};\sigma \right)=1-\exp\left(-R_{\mathrm{res}}^{2}/2\sigma^{2}\right)$ of its total power $P_{0}$ inside the tube. The antinode pressure amplitude at fixed power is

(1) $$P_{m}\left(R_{\mathrm{res}};\sigma \right)=P_{\max}\left(R_{\mathrm{res}}\right)\cdot \lambda \left(R_{\mathrm{res}};\sigma \right),$$

where $P_{\max}\left(R_{\mathrm{res}}\right)$ is the asymptotic pressure approached by an infinitely thin beam. The closed form factorizes the dependence on $R_{\mathrm{res}}$ and σ and, after normalization, collapses the joint sweep onto a single envelope (Figure 3). Two quantities must be distinguished. The experimental optical beam has a measured $1/e^{2}$ waist diameter of 2.15 mm, whereas the parametric acoustic model uses an effective Gaussian volumetric heat-source diameter $2w_{0}=1.6$ mm (σ=0.4 mm). The latter is a simulation input describing the deposited heat distribution, not a second measurement of the optical waist. For that simulated heat-source width, the antinode pressure is maximized at $R_{\mathrm{res}}=0.66$ mm. A tube this narrow is not acceptable because shrinking the radius raises the volume fraction of the viscous boundary layer and lowers *Q*. Moreover, 2.15 mm is a diameter rather than a radius: the measured waist radius is 1.075 mm, only 33.1% of the selected 3.25 mm tube radius. The Gaussian enclosure relation therefore indicates that more than 99.99999% of the beam power is contained within the tube at the waist. The selected radius is consequently governed by the joint pressure–loss–*Q*–frequency trade-off rather than by the minimum aperture needed to pass the beam.

### 3.2. Resonance Frequency and Quality Factor Trade-Off

**Figure 4:**
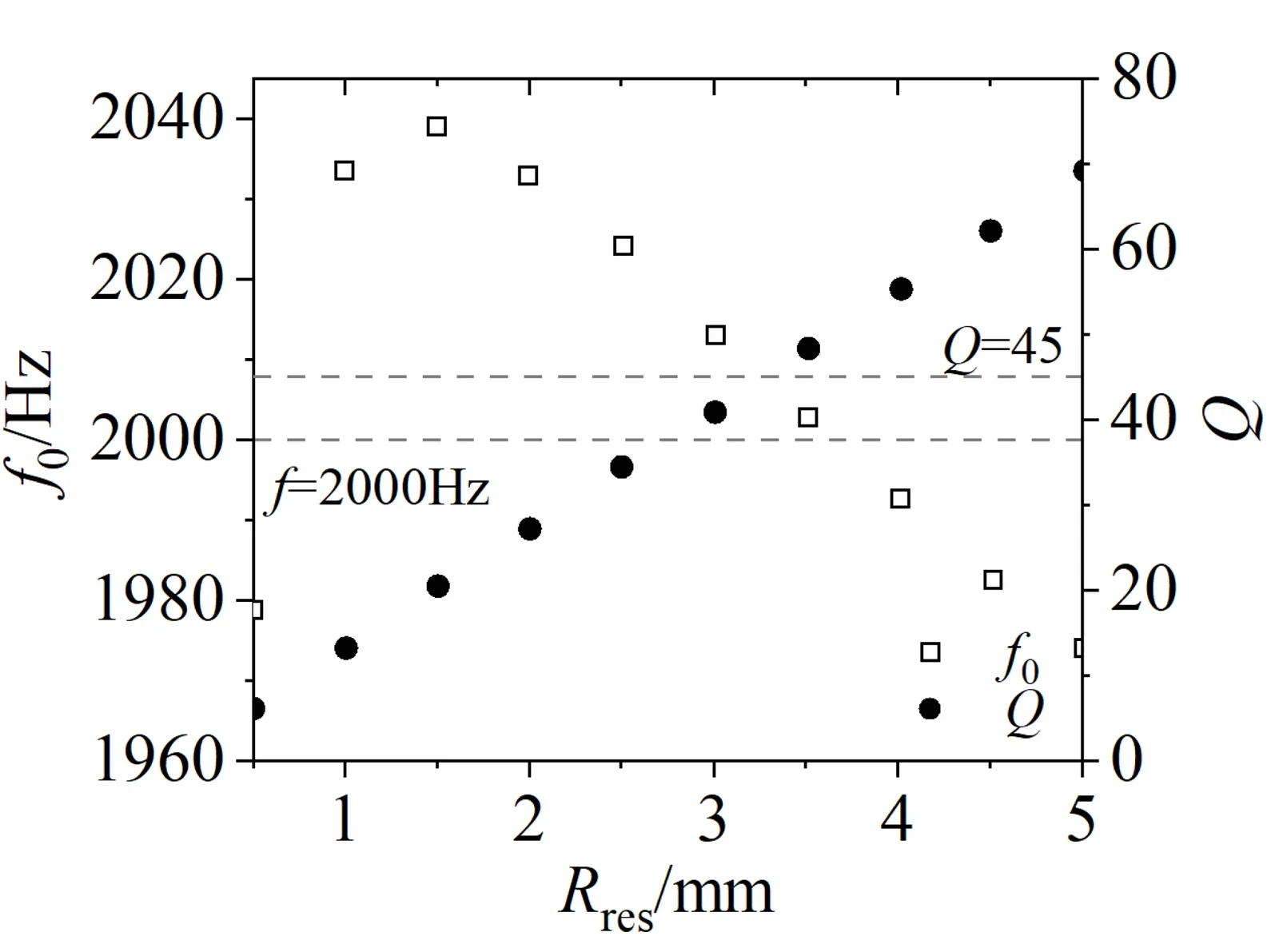
Resonance frequency $f_{0}$ and quality factor *Q* versus resonance tube radius; the operating radius of 3.25 mm balances the antinode pressure against the low-frequency noise floor.

The resonance frequency and *Q* of the first longitudinal mode depend on the tube radius through the acoustic mass loading and the viscous dissipation. Figure 4 shows the parametric dependence: the resonance frequency peaks near $R_{\mathrm{res}}=1.5$ mm and then declines with increasing radius, while the quality factor rises monotonically with radius. Field mechanical vibration and flow noise are broadband, with the highest energy density at low frequencies; the resonance must sit above this floor to protect the signal-to-noise ratio, while a low *Q* broadens the resonance and admits wideband noise through the cell’s band-pass action. The two requirements, together with the antinode pressure of Equation (1), select $R_{\mathrm{res}}=3.25$ mm as the operating radius, with a beam-defined pressure lower than the unconstrained maximum at 0.66 mm but with *Q* held above 40 and $f_{0}$ above 1600 Hz.

### 3.3. Longitudinal Dimension and Quarter-Wavelength Buffer Condition

The buffers transmit external acoustic noise into the resonance tube. The transmitted noise of wavelength λ is minimized by setting the buffer length to $L_{\mathrm{buf}}=\lambda /4$; for the same-frequency case, setting $L_{\mathrm{buf}}=0.5L_{\mathrm{res}}$ places this null at the cell’s resonance and preserves the half-wave longitudinal mode. Parametric sweeps over the buffer and tube dimensions show that lengthening the tube at fixed buffer size, or enlarging the buffer at fixed tube length, both raise the antinode pressure but lower the resonance frequency and *Q*. A buffer of length 50 mm and radius 20 mm with a 100 mm tube keeps the resonance above 1600 Hz and *Q* above 40. A two-dimensional axisymmetric model and a three-dimensional full model agree closely, confirming the axisymmetric reduction.

Substituting the radial and longitudinal values into the three-dimensional full model, the acoustic parameters before and after optimization are summarized in Table 1: the resonance frequency falls from 1929 Hz to 1612 Hz, *Q* from 88.40 to 44.03, and the antinode pressure rises from 0.4710 Pa to 0.7909 Pa, giving a conversion amplification of 0.7909/0.4710≈1.6. The optimization exchanges about half of the original *Q* for roughly 1.6 times the antinode pressure, which is the compromise that the hard constraints $f_{0}>1600$ Hz and Q>40 require.

## 4. Differential Extraction and Lossless Pickup of the Standing-Wave Signal

Differential Helmholtz cells already provide effective common-mode rejection by subtracting the outputs of matched microphones [10,11]; the present longitudinal architecture uses the same general differential principle and is not proposed as a universally superior noise-rejection topology. It was selected because its straight tubes accommodate the collimated optical path, provide accessible pressure antinodes at the tube centers, and permit direct microphone insertion at an antinode. Coupling the two tubes through shared buffers adds a design-specific feature: the first longitudinal resonance becomes an anti-phase/in-phase eigenmode pair whose separation and *Q* can be tuned through the tube spacing. The differential arrangement changes the modal structure of the cell, while the direct-insertion mount changes the impedance presented to that structure. This section treats the two as a single signal-transfer chain.

### 4.1. Differential Extraction Through Buffer-Coupled Modal Splitting

**Figure 5:**
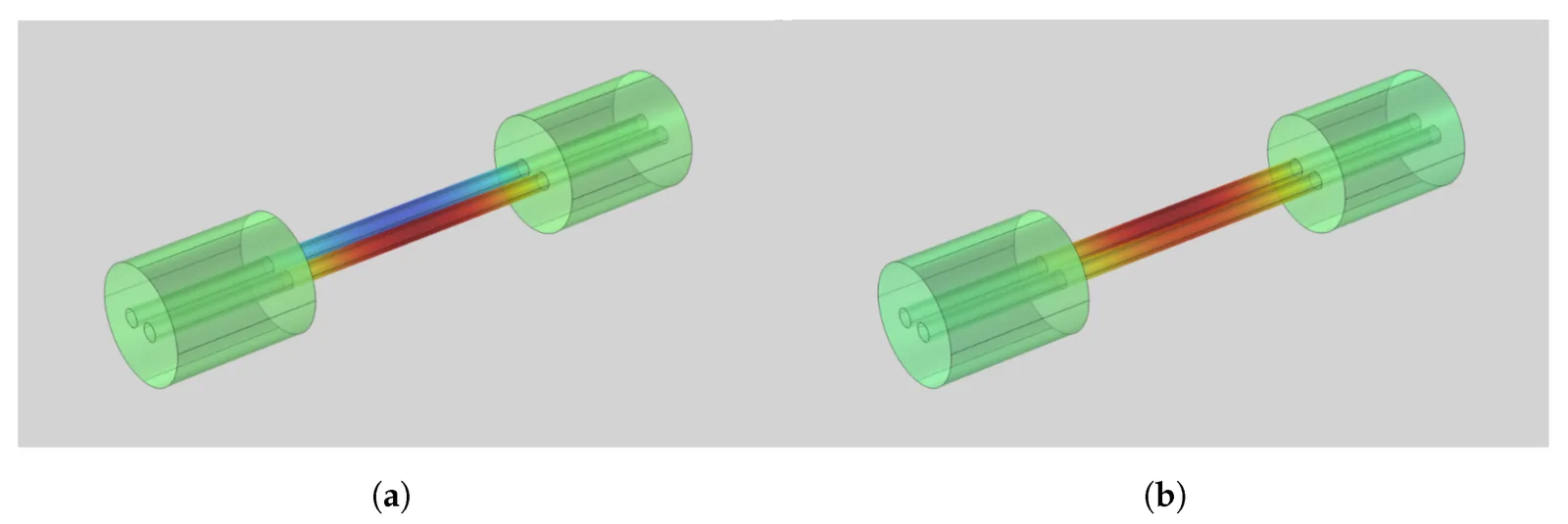
Modal splitting of the dual-tube system: (**a**) anti-phase mode at 1597 Hz; (**b**) in-phase mode at 1621 Hz.

Placing a second, geometrically identical tube 5 mm from the optimized tube (the working tube A) and coupling the two through the shared buffer volumes turns the single cell into a dual-tube system. The coupling splits the first longitudinal resonance into two eigenmodes present in both tubes: a low-frequency anti-phase mode at 1597 Hz, in which the two tube pressures differ by $180^{\circ }$, and a high-frequency in-phase mode at 1621 Hz, in which they are equal (Figure 5). The sensor is operated at the anti-phase resonance; the in-phase resonance is the companion eigenmode of the coupled structure, not a second simultaneous measurement channel. Dual-mode behavior in differential resonant cells is not new; Sherstov and Chetvergova [23] experimentally observed the ring longitudinal modes of differential photoacoustic detectors of Miklos and Kapitanov, and coupled in-phase and anti-phase eigenmodes of dual-tube systems have been characterized for capillary-loaded microphones [24]. The present contribution is the quantitative account of how the two split modes, the two *Q* values, and the differential and common-mode amplitudes evolve with tube-center spacing, together with the geometric rule that the spacing should equal the buffer radius.

**Figure 6:**
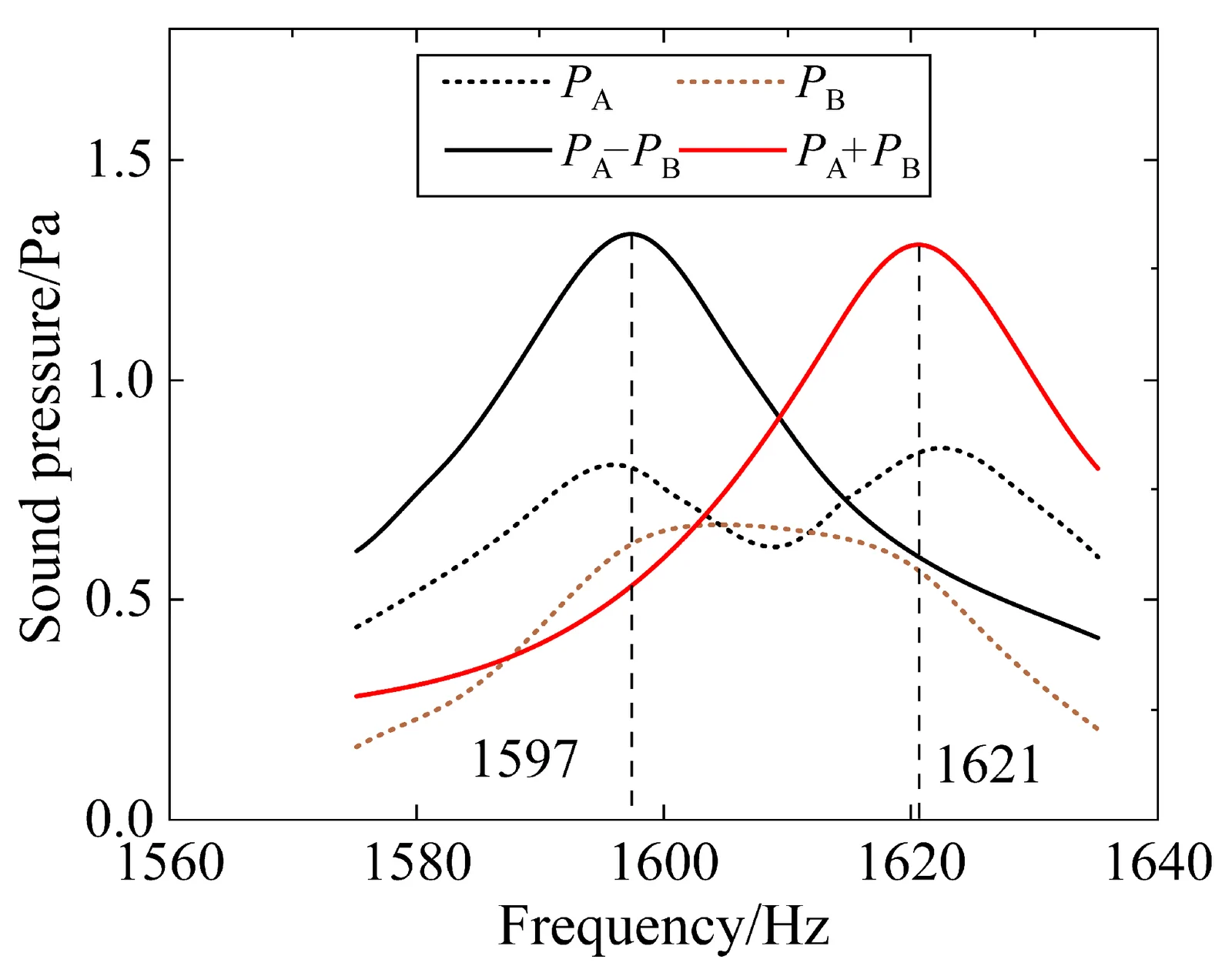
Amplitude–frequency responses of the dual-tube system, showing the sum and difference signals that drive the differential extraction.

The amplitude–frequency responses of the two tubes and of their sum and difference are shown in Figure 6. The working tube shows a double-peaked response, while the reference tube shows a single broad peak; both the sum and the difference are single-peaked, with peaks approaching twice the single-tube extremum. Because the two split modes are close in frequency, both are excited when the modulation frequency approaches this band, which is what broadens the working-tube response. The difference peak, corresponding to the anti-phase mode, sits at the lower frequency and is therefore the larger, consistent with the inverse-square frequency scaling of the resonant amplitude.

Ambient low-frequency vibration and flow noise have wavelengths on the order of meters, three orders of magnitude larger than the millimeter-scale tube separation, so they excite nearly in-phase pressure fluctuations in the two tubes. Taking the difference of the two antinode pressures cancels this common-mode disturbance and yields a usable simulated signal about twice that of a single tube, without any increase in laser power. This factor of two is the ratio of the difference peak to the single-tube extremum read from Figure 6, and it originates in the lower frequency of the anti-phase mode rather than in noise rejection alone. The modal-amplitude effect is specific to the present coupled longitudinal geometry and does not imply that differential Helmholtz cells lack common-mode rejection. A controlled head-to-head experiment would be required to establish general superiority over a differential Helmholtz configuration.

**Figure 7:**
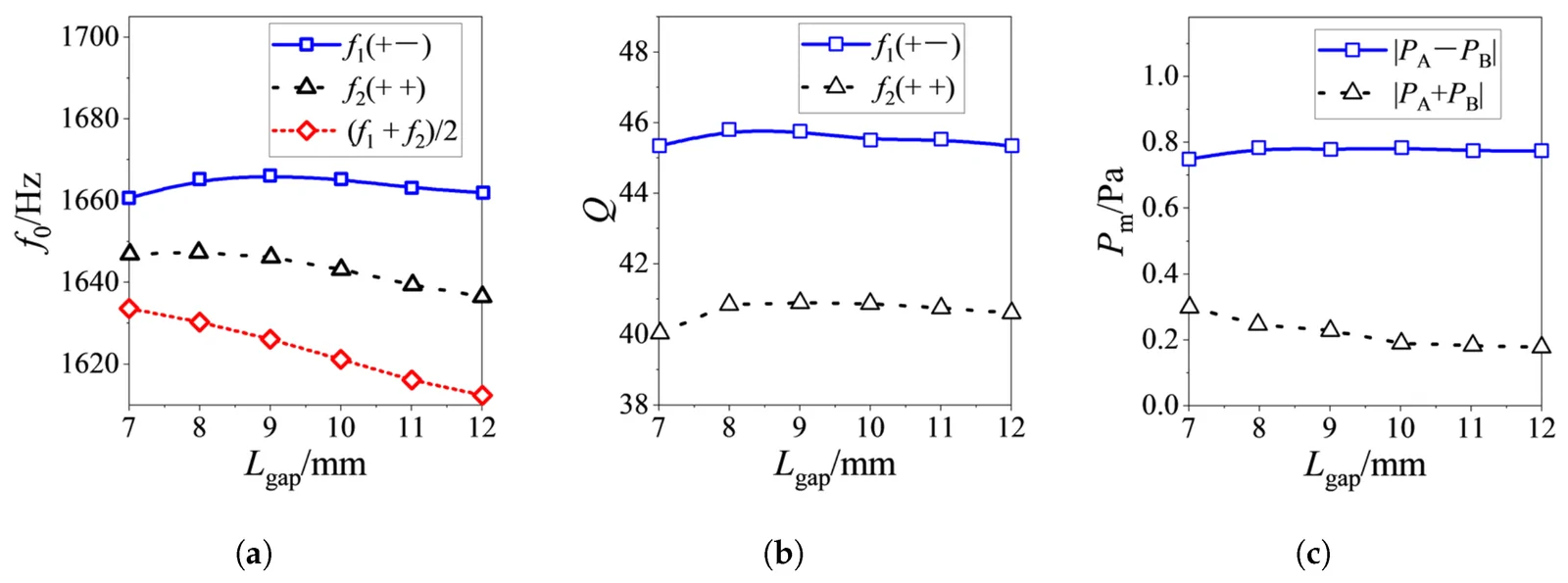
Tube-center spacing dependence: (**a**) anti-phase and in-phase frequencies; (**b**) anti-phase and in-phase quality factors; (**c**) differential and common-mode signal amplitudes.

The center-to-center spacing $L_{\mathrm{gap}}$ sets the strength of the acoustic coupling through the buffer. The split frequency, the two *Q* values, and the differential signal amplitude are interdependent, so $L_{\mathrm{gap}}$ is selected by a joint trade-off rather than by a single-objective optimization. The three dependencies are shown in Figure 7. The anti-phase frequency falls monotonically with $L_{\mathrm{gap}}$, while the in-phase frequency rises and then falls, so the average is roughly constant and the split widens as the coupling weakens. The anti-phase *Q* reaches a maximum and the in-phase *Q* a minimum at $L_{\mathrm{gap}}$ equal to the buffer radius, and the differential signal amplitude peaks at the same spacing while the common-mode amplitude falls monotonically. The spacing equal to the buffer radius is adopted. Importantly, this spacing is not a new degree of freedom: it is fixed by the same buffer geometry that sets the longitudinal scale, so the introduction of the differential arrangement does not enlarge the design space.

### 4.2. Acoustic Load of the Pickup and Impedance-Matched Insertion

**Figure 8:**
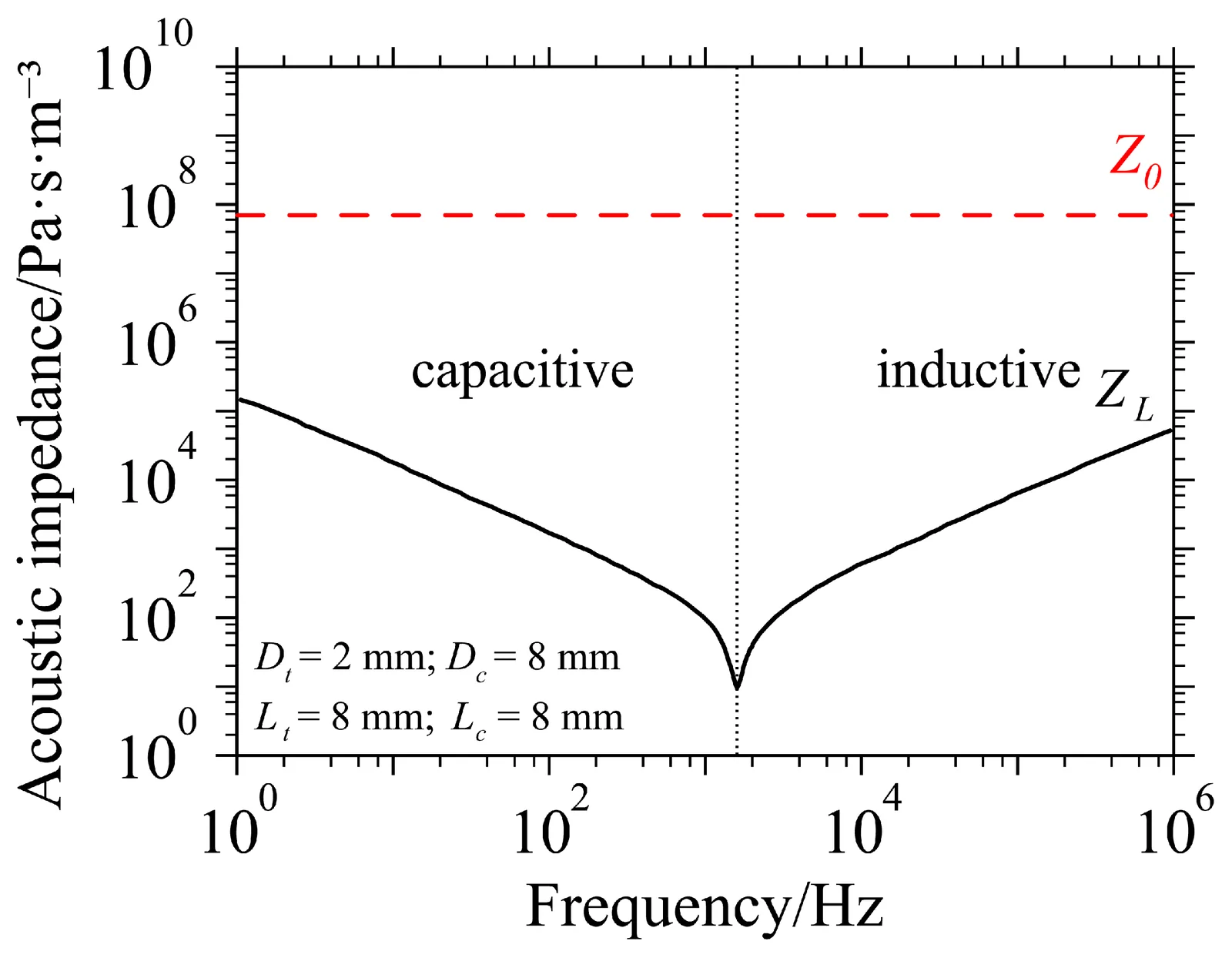
Pickup impedance $Z_{c}$ versus the tube characteristic impedance $Z_{0}$; the low-impedance dip at the pickup resonance shunts the antinode pressure.

The antinode sits at the axial center of the tube, and the conventional way to reach it is a narrow duct rising from the tube to a microphone cavity. The duct-plus-cavity geometry is acoustically a Helmholtz resonator. A transmission-line model of the resonance tube loaded by the pickup maps the acoustic problem onto a telegrapher’s-equation analogue, with the resonance tube as a transmission line of characteristic impedance $Z_{0}$, the buffers as low-impedance terminations, and the pickup as a lumped impedance $Z_{c}$ shunted across the line at its center. The pickup impedance $Z_{c}$, plotted in Figure 8, falls far below $Z_{0}$ at the cell’s resonance. A low shunt impedance at the center of the line diverts the axially propagating acoustic energy into the pickup, so the antinode pressure cannot accumulate and the output collapses.

**Figure 9:**
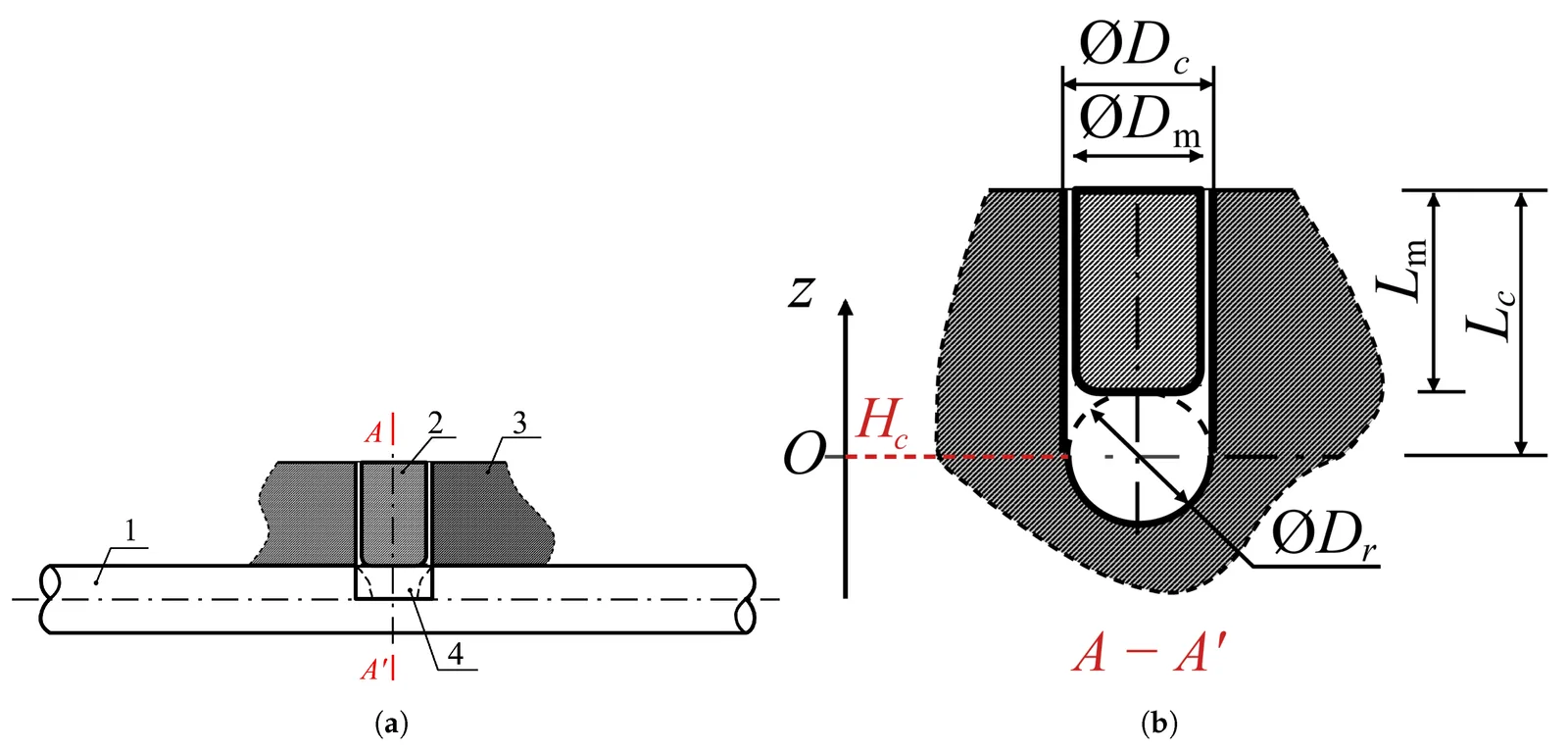
Direct-insertion pickup structure: (**a**) front view, showing the resonance tube (1), microphone (2), surrounding mount (3), and the opening below the sensing face (4); (**b**) side view, with the microphone sensing face tangent to the tube inner wall.

The impedance mismatch is removed by eliminating the duct. A slot of diameter slightly larger than the microphone is machined downward from the top of the tube, and the microphone is inserted so that its sensing face is geometrically tangent to the tube’s cylindrical surface (Figure 9). If the face stands proud of the inner wall, a residual capacitive cavity forms and lowers the load impedance; if it intrudes, it scatters the beam. Parametric sweeps on the slot diameter and depth show that the resonance frequency and *Q* are nearly insensitive to the depth, while the sensed pressure is maximal when the slot bottom is level with the tube axis ($H_{c}=0$).

The differential structure acts at the level of the modal structure and separates the signal from the noise; the direct-insertion pickup acts at the level of the impedance and couples the separated pressure losslessly to the microphone. Together they form a complete chain from the standing wave to the demodulatable electrical signal.

## 5. Experimental Verification and Discussion

**Figure 10:**
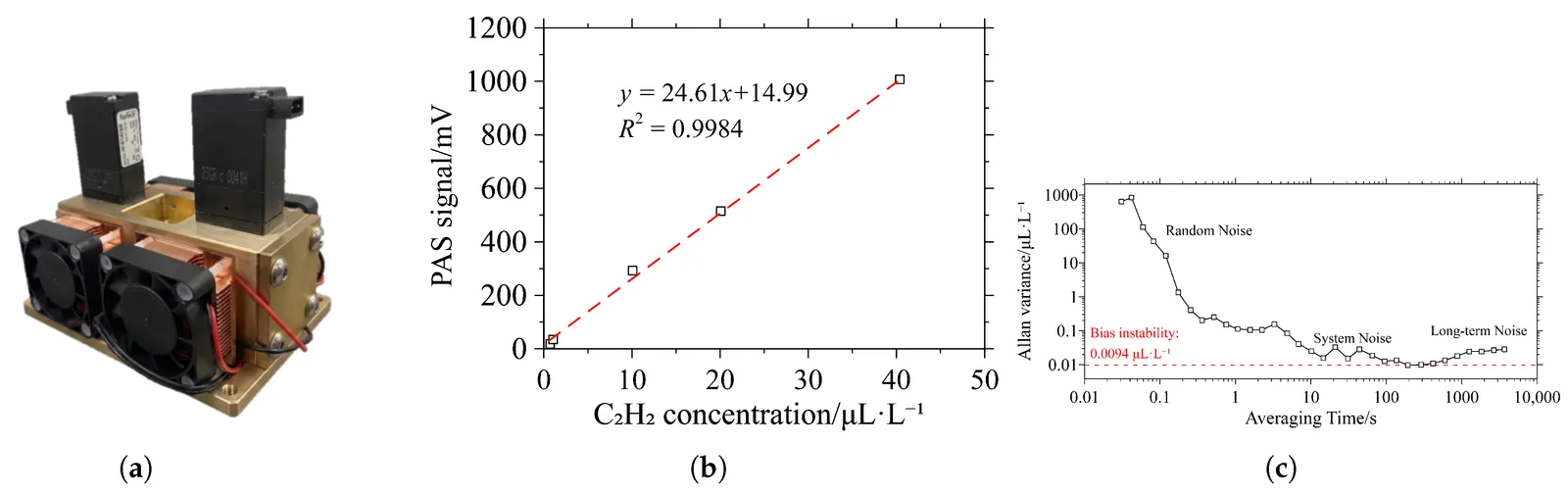
Experimental characterization of the fabricated cell: (**a**) photograph of the cell; (**b**) photoacoustic signal versus C_2_H_2_ concentration (24.6 mV/ppm, $R^{2}=0.9984$); (**c**) minimum Allan deviation of 9.4 ppb at 300 s, corresponding to a 3σ limit of detection of 28.2 ppb.

A cell was machined to the optimized geometry, integrating the temperature-control unit and the gas valves (Figure 10a). With the wavelength-modulation parameters of the platform [25], the C_2_H_2_ response is linear with a sensitivity S=24.6 mV/ppm and $R^{2}=0.9984$ (Figure 10b). The minimum Allan deviation at a 300 s integration time is $\sigma_{c}=9.4$ ppb (Figure 10c) [26,27]. Applying the requested 3σ/sensitivity criterion gives

(2) $${\mathrm{LOD}}_{3\sigma }=\frac{3\sigma_{V}}{S}=3\sigma_{c}=28.2\;\mathrm{ppb},$$

where $\sigma_{V}=S\sigma_{c}$ is the voltage-domain standard deviation corresponding to the concentration-domain Allan deviation.

Selectivity was evaluated experimentally with a prepared gas sample using the same photoacoustic cell. The sample contained C_2_H_2_ together with the principal hydrocarbon and carbon-dioxide interferents encountered in dissolved-gas analysis: the gas-phase concentrations of C_2_H_4_, C_2_H_6_, and CH_4_ were each above 150 μL/L, and that of CO_2_ was above 5000 μL/L. The measured C_2_H_2_ concentration was compared with the reference value obtained by laboratory gas chromatography(ZF-301Q, Henan Zhongfen Instrument Co., Ltd., Shangqiu, China). In this mixed-gas sample, the absolute C_2_H_2_ error was 0.09 μL/L and the relative error was 4.09%. Because the experiment includes wavelength-selective excitation and photoacoustic readout with the complete gas-phase measurement configuration, it evaluates system-level measurement selectivity rather than the acoustic cell in isolation. The result supports practical rejection of the tested interferents under the stated conditions, but does not imply complete independence from gas-matrix composition. Previous work has shown that water vapor and oxygen can alter methane photoacoustic signals through kinetic-cooling effects [28]; whether an analogous matrix dependence affects the present acetylene measurement requires a dedicated test.

The 9.4 ppb minimum Allan deviation and the corresponding 28.2 ppb 3σ detection limit must be read against the methods that produced them. The acoustic core of the cell was jointly optimized, the transducer was a plain condenser microphone, and no multivariate post-processing was applied. The sub-parts-per-billion results of recent work were obtained at the cost of additional post-processing (such as the partial-least-squares regression of [12]), alternative transducers (quartz tuning forks, microphone arrays [18]), or optical-path multiplication (multipass cavities [16,19]); the present result is obtained at the cost of the cell structure. The performance increment comes from the design rather than from the addition of further devices.

The source of that increment lies in a set of trade-offs that the design must navigate. The antinode pressure favors a small tube radius and a low resonance frequency; the quality factor favors a larger tube radius to reduce the relative importance of the viscous boundary layer; and the resonance frequency must remain above the low-frequency noise floor. A high *Q* brings resonance gain but narrows the bandwidth and increases sensitivity to temperature drift. Established analytical rules, finite-element models, design-of-experiments methods, and NSGA-II searches can all describe parts of this design space [6,7,8,9,29]. The purpose of the present sequence is not to replace those approaches but to make the selected compromise traceable for this dual-tube geometry.

Within that established framework, the beam–tube coupling is encoded here in the closed form $\lambda \left(R_{\mathrm{res}};\sigma \right)\cdot P_{\max}\left(R_{\mathrm{res}}\right)$, the longitudinal scale in the quarter-wavelength buffer condition, and the pickup in the transmission-line model of the resonance tube loaded by the microphone cavity. With these criteria in place, the system-specific compromise is selected under the constraints $f_{0}>1600$ Hz and Q>40 while maximizing antinode pressure. The value of this formulation is the integration of the criteria and the explicit connection to the fabricated geometry, rather than a claim that coupled parameter effects have not been modeled previously.

The same scope applies to the differential section. Common-mode rejection is shared with conventional differential Helmholtz cells [10]; it is not unique to the present architecture. The design-specific contribution is the quantitative mapping of the split, the two *Q* values, and the differential and common-mode amplitudes onto tube-center spacing, and the rule that the spacing equal the buffer radius. The measured quantity is obtained at the anti-phase resonance; the in-phase resonance is its coupled companion rather than a required second operating channel. The approximately twofold simulated differential amplitude arises from the lower-frequency anti-phase mode and is distinct from common-mode rejection. The straight optical path and accessible central antinodes also allow the direct-insertion pickup used here, but a controlled comparison would be required before claiming general superiority over a differential Helmholtz cell.

In the pickup section, the transmission-line model exposes what the acoustic equations hide: the microphone cavity is not a passive sensor but a load on the standing wave, and unless the load impedance is at least comparable to the line impedance, the standing wave is disturbed. The early recognition that the microphone impedance should be matched to the resonator [22] sits in the same conceptual lineage, but the lumped Helmholtz framework cannot describe the distributed loading of a longitudinal standing wave by a microphone cavity, which is the regime of interest here. The direct-insertion mount moves the machining tolerance from the duct to the slot bottom, a cost that the cell absorbs in exchange for the recovered antinode pressure. That this exchange is feasible at all reflects the interdependence of the design stages: a change in one place leaves a mark in another, and the design is meaningful only as a whole.

## 6. Conclusions

Building on established PA-cell design methods, this work applies a system-specific coupled optimization to a differential longitudinal resonator for trace acetylene detection. The beam–tube relation and quarter-wavelength buffer condition set a tube radius of 3.25 mm and a buffer of half the tube length under the constraints $f_{0}>1600$ Hz and Q>40, giving a simulated three-dimensional conversion amplification of 1.6. The dual-tube arrangement has a 1597 Hz anti-phase mode and a 1621 Hz in-phase companion mode; the sensor operates at the anti-phase resonance, where differential readout rejects spatially coherent common-mode noise and produces approximately twice the single-tube simulated amplitude. A direct-insertion microphone mount avoids the low-impedance shunt of a ducted pickup. The fabricated cell delivers a C_2_H_2_ sensitivity of 24.6 mV/ppm ($R^{2}=0.9984$), a minimum Allan deviation of 9.4 ppb at 300 s, and a corresponding 3σ limit of detection of 28.2 ppb. The prepared mixed-gas sample gives a C_2_H_2_ relative error of 4.09%, providing experimental evidence of system-level selectivity under the tested conditions.

## Figures and Tables

**Table 1:** Acoustic parameters before and after coupled multi-parameter optimization (three-dimensional full model).

Configuration	$f_{0}$ (Hz)	*Q*	$P_{m}$ (Pa)
Before	1929	88.40	0.4710
After	1612	44.03	0.7909

## Data Availability

The data presented in this study are available on request from the corresponding author.

## Abbreviations

The following abbreviations are used in this manuscript:

PAS	photoacoustic spectroscopy
DGA	dissolved gas analysis
*Q*	quality factor
C_2_H_2_	acetylene
SNR	signal-to-noise ratio
